# Feasibility of Motor Imagery Training for Children with Developmental Coordination Disorder – A Pilot Study

**DOI:** 10.3389/fpsyg.2017.01271

**Published:** 2017-07-26

**Authors:** Imke L. J. Adams, Bouwien Smits-Engelsman, Jessica M. Lust, Peter H. Wilson, Bert Steenbergen

**Affiliations:** ^1^Behavioural Science Institute, Radboud University Nijmegen, Netherlands; ^2^Department of Health and Rehabilitation Sciences, Faculty of Health Sciences, University of Cape Town Cape Town, South Africa; ^3^School of Psychology, Australian Catholic University, Melbourne VIC, Australia; ^4^Centre for Disability and Development Research, Australian Catholic University, Melbourne VIC, Australia

**Keywords:** MI training, action observation, cognitive orientation to daily occupational performance, CO-OP, DCD, internal model

## Abstract

Children with Developmental Coordination Disorder (DCD) experience movement difficulties that may be linked to processes involved in motor imagery (MI). This paper discusses recent advances in theory that underpin the use of MI training for children with DCD. This knowledge is translated in a new MI training protocol which is compared with the cognitive orientation to daily occupational performance (CO-OP). Children meeting DSM-5 criteria for DCD were assigned to MI (*n* = 4) or CO-OP (*n* = 4) interventions and completed nine treatment sessions, including homework exercises. Results were positive, with two children in the MI group and three in the CO-OP group improving their m-ABC-2 score by ≥ 2 standard scores, interpreted as a clinically meaningful change. Moreover, all children and parents noticed improvements in motor skills after training. This is the first study to demonstrate the feasibility of a theoretically principled treatment protocol for MI training in children with DCD, and extends earlier work.

**Trial registration:** The complete trial is registered at the Dutch trial register, www.trialregister.nl (NTR5471). http://www.trialregister.nl/trialreg/admin/rctview.asp?TC=5471

## Introduction

Children with Developmental Coordination Disorder (DCD), representing about 5–6% of the child population, experience movement difficulties that affect their participation in daily activities (Barnhart et al., 2003; Gaines et al., 2008). These difficulties have developmental consequences beyond motor function, and place the child at a significant risk for social, psychological, and health-related issues, extending into adulthood (Hellgren et al., 1993; Rasmussen and Gillberg, 2000). Although for the population of children with DCD, treatment programs are available (such as the cognitive orientation to daily occupational performance (CO-OP) (Barnhart et al., 2003; Sangster et al., 2005) or neuromotor task training (NTT) (Schoemaker et al., 2003; Schoemaker and Smits-Engelsman, 2005; Ferguson et al., 2013), our understanding of the developmental and neurocognitive mechanisms involved is still evolving (Wilson et al., 2013; Adams et al., 2014). As this knowledge unfolds, so too will our capacity to refine intervention practices (Wilson et al., 2016b).

Motor performance in children with DCD can be characterized by slow, effortful, inaccurate, and ill-coordinated movements that are overly dependent on visual feedback (Deconinck et al., 2006; Wilson et al., 2013). Importantly, a meta-analysis suggests that deficits in predictive motor control and perceptual-motor coupling may explain these issues in motor coordination and skill learning (Wilson et al., 2013). Taking up this lead more specifically, a review by Adams et al. (2014) examined predictive motor control (an aspect of internal modeling) in DCD and provided a number of new insights into the (neurocognitive) mechanisms that may underlie the disorder. Studies using a range of experimental paradigms including covert orienting of visuospatial attention, imagined or simulated pointing, mental rotation of limb- versus object-based stimuli, predictive control of eye movements, grip force and anticipatory postural adjustments, and studies on the rapid online control of reaching movements indicate that children with DCD have a deficit in motor prediction and online control (Wilson et al., 2013), termed the internal modeling deficit (IMD) hypothesis (Wilson and Butson, 2007).

Internal modeling is a fundamental concept in motor control and learning (Wolpert, 1997; Jeannerod, 2001, 2006). Internal forward (or predictive) models contribute to volitional control by anticipating the sensory consequences of a given movement. In the case of DCD, the IMD hypothesis states that these children have difficulties generating or implementing predictive models of action leading to incomplete planning of a forthcoming action and a concomitant over-reliance on slower feedback-based control. This is shown by a reduced ability to imagine egocentric transformations of the body, as in mental rotation tasks using body-related stimuli (Deconinck et al., 2009; Williams et al., 2013), or explicit imagery tasks that require sequential movements to targets of different size, wherein the customary speed-accuracy trade-off seen in real movements is not observed (Lewis et al., 2008; Ferguson et al., 2015). Perhaps even more compelling is recent data showing poor prospective planning of target-directed movements in DCD (van Swieten et al., 2010; Adams et al., 2016a). While grasp orientations are generally planned with a forward view to end-state comfort (Rosenbaum et al., 1990), children with DCD are less able to plan prospective actions on this basis, particularly for more complex movements (e.g., grasping an object for subsequent insertion in a tight recess, or planning a sequence of movements that differ in terms on their onward intentions (e.g., to place or to throw) (Wilmut et al., 2013).

Recent research in adults with acquired brain damage (Ertelt et al., 2007; Page et al., 2007; Zimmermann-Schlatter et al., 2008; Pelosin et al., 2010; Kim and Lee, 2013) and studies in motor impaired children (Wilson et al., 2002, 2016a) support two techniques that target internal modeling: motor imagery (MI; internal rehearsal of a future motor action without overt motor output) and action observation (AO; observation of a to-be-learned action performed by someone else). MI and AO can be considered two sides of the same coin (namely motor simulation), MI being internally simulated motor action and AO being externally modeled motor action (Vogt et al., 2013). We already know that MI and AO play a role in learning and re-learning complex motor tasks and share common neurophysiological networks with internal modeling (Jeannerod, 2001). More recent data reinforce the point of substantial overlap on a neuroanatomical basis within the mirror neuron system (Jeannerod, 2006; Gatti et al., 2013; Vogt et al., 2013).

In 2011, Schuster and colleagues reviewed MI training studies for different disciplines. Successful elements in training were individual, supervised sessions added after physical practice. Of the 133 MI training studies that were reviewed by Schuster et al. (2011), only nine focused on participants under 18 years of age (e.g., Jarus and Ratzon, 2000; Taktek et al., 2004; Smith et al., 2007). In a review by Spruijt et al. (2015) it is concluded that MI ability in typically developing children improves steadily over childhood but approaches adult levels by mid-to-late adolescence. These developmental trends supported the view that MI training is feasible for pediatric rehabilitation in typically developing children as young as 5 years of age. The few experimental studies of AO/MI training in typically developing children (e.g., Atienza et al., 1998; Coelho et al., 2007; Guillot et al., 2015) and children with developmental motor disorders have shown great promise (Wilson et al., 2002, 2016a; Buccino et al., 2012; Sgandurra et al., 2013). For example, Guillot et al. (2015) found that embedding MI during a high intensity intermittent tennis training for typically developing children enabled the development of physical fitness and the preservation of stroke performance. For children with developmental motor disorders MI training has also been used. For example, in a pilot study, Buccino et al. (2012) had children with motor problems as a consequence of cerebral palsy (6–11 years) watch video excerpts of a specific daily action requiring the use of the arms and/or hands (i.e., grasping an object, using a pencil, playing with Lego) followed by execution of the same movement for 2 min. After treatment this group performed better on a test that measures the quality of upper limb motor functions [the Melbourne Assessment Scale (Randall et al., 2001)] than a control group receiving no AO training but instead observed videos with no specific motor content. In a group of children with mild to moderate DCD, Wilson et al. (2002) showed that a computer-based MI training (including AO elements) regime improved the level of movement skill, and showed comparable effects to physical therapy. This finding was replicated recently, in a cohort screened rigorously for DCD (Wilson et al., 2016a). In sum, converging evidence from behavioral and neuroimaging studies of motor control and action in DCD, adult neuropsychology, mainstream neuroscience of motor control in adults, and existing data on the effects of MI training across populations, suggest that MI is a prime modality that may serve motor intervention for children motor problems.

Currently, task-oriented approaches such as CO-OP are often used by occupational and physical therapists to treat children with DCD (Miller et al., 2001; Smits-Engelsman et al., 2013). The CO-OP approach is based on cognitive behavior modification theories, in particular the verbal self-instruction strategy developed by Meichenbaum (1997). During a CO-OP intervention, a child learns this self-instruction strategy, which enables the child to identify why the performance was not successful, and to invent and execute plans to correct his/her task performance (the ‘goal-plan-do-check’ strategy) (Barnhart et al., 2003). It is based on the belief that when a child guides himself through a problem-solving task by talking aloud, he/she learns to regulate behavior by learning how to identify a goal, develop a plan and evaluate the success of that plan (Sangster et al., 2005). Several studies have shown that the CO-OP intervention is effective in obtaining the goals chosen by children with DCD (Miller et al., 2001; Polatajko et al., 2001; Corcoran et al., 2005; Zwicker et al., 2015).

In this paper, we present the results of a multiple case study comparing MI with CO-OP training for children that meet the clinical criteria for DCD (DSM-V). The CO-OP training was considered the usual care for children with DCD since it is recommended as one of the treatment options in the EACD guidelines (Blank et al., 2012; Smits-Engelsman et al., 2013) Importantly, to enhance the application of MI training, we use a recently published, systematic protocol for MI training (Adams et al., 2016b). In line with the recommendation of Vogt et al. (2013), the protocol combines both MI and AO, where a video model performing the trained motor skill always preceded the imagery element. To better assess the feasibility and effectiveness of the training protocol we use a multi-method evaluation combining measures of movement competency, qualitative reports from children and their parents, and self-reports from therapists about their experience of using the protocol. Our specific objectives were:

(1) Study individual changes of motor skills after an MI and CO-OP training in children with DCD.
(2) Assess experiences of participating children and parents via examination of perceived improvement of motor skills by both, children and parents and by examining if children enjoyed the training method.
(3) Assess the ease of implementation of the protocol, by examining therapists’ experiences.

## Materials and Methods

### Participants

Eight children (three boys, five girls) aged 7–12 years were included in this pilot study. Children were allocated to either the MI (*n* = 4, one left-handed) or CO-OP (*n* = 4, all right-handed) group. Mean age was 9.5 (range 7.9–12.1) and 9.4 years (8.2–12.0) for the MI group and CO-OP group, respectively.

The children with DCD were recruited through pediatric physical therapists (PPTs) and occupational therapists (OTs) who were trained to deliver either the MI or CO-OP training (see also Adams et al., 2016b). Included children all met the four DSM-5 diagnostic criteria for DCD (American Psychiatric Association, 2013): (1) m-ABC-2 (Henderson et al., 2007), Dutch validation (Smits-Engelsman, 2010) total score ≤ 16th %tile or component score ≤ 5th %tile (criterion A DSM-5), (2) referred to a PPT or OT for motor training and the DCD Questionnaire (DCDQ) (Schoemaker et al., 2008) was used to further assess interference of the motor impairment with daily activities and/or academic achievement (criterion B DSM-5), (3) onset of symptoms in the early developmental period as evidenced by their referral to a center for motor training between the ages of 7–12 years (criterion C DSM-5), and (4) absence of any medical condition that could cause the motor impairment and IQ ≥ 70. This was checked using a health questionnaire, completed by parents/caregivers. A diagnosis ADHD was not an exclusion criterion, involved therapists determined whether the child had enough attentional capacity to be engaged in the MI or CO-OP training.

Pediatric physical therapists and OTs that provided the MI (*n* = 3) or CO-OP training (*n* = 4) for this study were aged 29–44 years. Most therapists provided the treatment for one child in this study, only one therapists provided therapy for two children in this study (case 1 and 3 MI training – see **Table 1**). Three PPTs were working in private practices, and two PPTs worked in a rehabilitation centre. The two OTs that provided the training, worked in a rehabilitation centre.

**Table 1:** Participants’ characteristics and trained motor skills.

Subject	Age (range in years)^∗^	DCDQ-score	m-ABC-2 percentile score (T0)	Imagined and trained motor skills	
**MI-training**					
1	11–12	24	9.0	Running: - running, grab an object and run back - slalom forward and backward - running toward an unpredictable object (square ball) - playing tag	Throwing and catching: - underhand throw and catch – both hands and one hand - throwing upward, catching and bounce - overhand throw and catch – both hands and one hand - throwing at a target and catch
2	8–9	28	0.1	Jumping a rope (turning the rope oneself): - pace of turning the rope - jumping forward - jumping high enough - turning the rope forward and backward	Jumping a rope (Jumping in): - pace of jumping - in-between jump - position where to jump - jumping with and without trampoline - lifting the legs high enough
3	8–9	56	5.0	Running: - running fast and decelerate and stop - slalom forward and backward - running toward and unpredictable object (square ball) - playing tag	Writing: - sitting correctly at the table - position of paper - holding the pencil - moving smoothly over the paper - writing neatly
4	7–8	26	0.5	Throwing: - underhand throw - overhand throw - throwing against wall and catch	Catching: - catching different balls - catching from different distances
**CO-OP training**					
1	11–12	32	1.0	Bicycling: - get the bike - get on the bike - moving the pedals forward - turning with the bike - stopping - get of the bike	Throwing and catching a basketball: - Aiming at basketball net - bounce the ball - Running toward basketball net and throw the ball
2	9–10	37	2.0	Jumping rope: - turning the rope oneself - pace of turning - pace of jumping - jumping at one place	Playing badminton: - serving underhand - hitting back underhand or overhand - walking backward
3	7–8	42	5.0	Tying shoe laces: - feet at chair or footstool - making a loop, and turning the other part around it	Eating with knife and fork: - sitting correctly at the table - holding knife and fork - position of elbows - use knife to push some food on the side of the fork
4	7–8	47	0.5	Bicycling: - position of pedals - balance only when moving - starting to cycle - turning - stop cycling and brake - get off the bike	Writing: - not writing too fast - writing letters with enough space between letters - writing neatly

*^∗^Because of anonymity of the participants, the age range in years is given instead of the exact age.*

### Training

Following our protocol, both the MI and CO-OP training were delivered for 9 weeks, with one training session per week lasting 45 min. Children also received a homework booklet and were required to practice four times per week for 10 min at home. Two self-selected skills of importance to the child were the focus of training. The Motor Coordination Questionnaire [MCQ, adapted from the ‘How Am I doing questionnaire’ (Calame et al., 2009, unpublished), also reported in Noordstar et al. (2017)], was completed by both the parents and the children (guided by the therapist). The MCQ helped to isolate those motor skills that were difficult (part A) and important for the child (part B).

#### Motor Imagery Training

The training protocol consisted of five parts: (a) discuss homework completed in the past week and determine the goal of the current session (10 min), (b) watch videos of a selected motor skill from 3rd-person perspective and 1st-person perspective, followed by mental rehearsal of this skill (10 min), (c) overt practice of the motor skill (10 min), (d) alternate mental rehearsal and overt practice of the motor skill, and compare and reflect on the two (10 min), (e) explain homework for the coming week, advise parents to motivate their child, and determine goals for the week ahead (5 min) – see also Adams et al. (2016b).

#### CO-OP Training

Cognitive orientation to daily occupational performance is expected to improve the knowledge of the task through cognitive strategy use (Polatajko and Mandich, 2004; Sangster et al., 2005) and is based on the verbal self-instruction strategy developed by Meichenbaum (1997). The training protocol consists of three main parts: (a) discuss homework from the previous week and determine the goal of the current session (10 min), (b) practice the selected motor skill using the Goal-Plan-Do-Check framework (30 min), (c) explain homework for coming week, tips for parents to motivate their child, and determine the goal of the week (5 min).

### Outcome Measures

All tests were performed by one trained assessor who was blind to group allocation at baseline (T0) and post treatment (T1).

#### Movement Assessment Battery for Children (m-ABC-2)

The score on the m-ABC-2 (Henderson et al., 2007 – Dutch validation: Smits-Engelsman, 2010) reflected the fine-motor skills, ball skills, and balance. The individual change score was the pre–post difference on m-ABC-2 standard scores. For the m-ABC-2 the smallest detectable difference (SDD 95%), regarded as clinically relevant (as reported in the manual) is two standard scores (Smits-Engelsman, 2010).

#### Experiences of Children and Parents

Parents were asked to fill in the MCQ about 16 motor skills before and after training. Prior to the start, they were asked how well their child performed these motor skills (part A), and rated the importance of each skill to their child (part B). Children completed the same questionnaire during the first training session. Following the training, parents and children were asked again to fill in this questionnaire, but instead of part B it was asked to indicate whether they thought that their (child’s) performance on these motor skills had improved or not (part C). Answers to part A and part B were filled out on a 5-point Likert scale. For part C of the MCQ, a 5-point scale was used from -2 (skill became much worse) to +2 (skill became much better). After the training, children were asked to fill in the Enjoyment Scale; a 5-point scale with smiley faces (0 no fun at all; 4 super fun) that has been developed by Jelsma et al. (2014).

#### Therapists’ Experiences

Based on contact between the researchers and the therapists before, during and after the training, experiences of the participating therapists are reported.

### Ethics Statement

This study was carried out in accordance with the recommendations of the Committee on Research Involving Human Subject of the region Arnhem-Nijmegen in the Netherlands with written informed consent from all subjects. Children’s parents gave written informed consent and children approved verbally in accordance with the Declaration of Helsinki. The protocol was approved by Committee on Research Involving Human Subject of the region Arnhem-Nijmegen in the Netherlands (protocol number 2013/463). The complete trial is registered at the Dutch trial register, www.trialregister.nl (NTR5471).

### Data Analysis

As this is a pilot study, we only present descriptive results. Median scores for each group are reported, as well as individual scores.

## Results

Age, DCDQ score and total percentile score on the m-ABC-2 before the training (T0) are displayed for each child in **Table 1**. One child in the MI training group had a ADHD diagnosis. The therapist in question judged this child’s attentional skills to be sufficient for the MI training. The different motor skills that were trained during the MI and CO-OP are also displayed in **Table 1**.

### Individual Change Scores on the m-ABC-2

**FIGURE 1:**
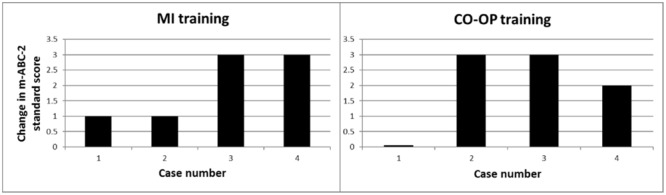
Individual (pre–post) change scores on the m-ABC-2. Positive values indicate improvement.

The individual change scores are displayed in **Figure 1**. Two children in the MI group and three children in the CO-OP group improved their m-ABC-2 total score with 2 or more standard scores.

### Experiences of Participating Children and Parents

**FIGURE 2:**
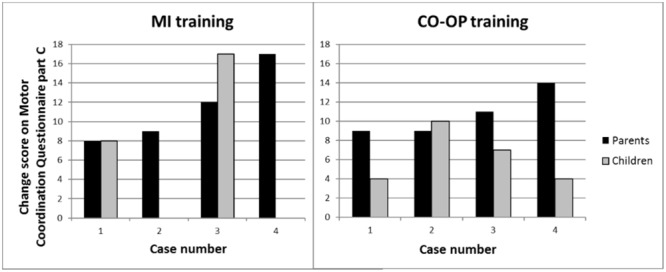
Perceived change of motor skills after the training of both parents and children (part C Motor Coordination Questionnaire). Positive values indicate improvement.

The results on part C of the MCQ are displayed in **Figure 2**. In the MI group parents reported a median increase of skills of 10.5 points (range 8.0–17.0), in the CO-OP group an increase of skills of 10.0 points (9.0–14.0). The children in the MI group reported a median increase of 12.5 points (8.0–17.0), in the CO-OP group an increase of 5.5 points (range 4.0–10.0). Data from part C of the MCQ of two children in the MI group were missing because these two therapists forgot to fill out the MCQ during the last treatment session.

Data from one child in the MI group on the Enjoyment Scale was missing. A median score of 3.0 (range 2.0–4.0) was reported in the MI group, and also median score of 3.0 (range 3.0–4.0) in the CO-OP group.

### Therapist’s Experiences

Therapists that provided the MI training to children (*n* = 3) were asked about their experiences with the training. They reported that it was hard to stick to the protocols guidelines, because they are used to combine several approaches (such as cognitive strategies from the CO-OP, or principles of motor control and motor learning from NTT). However, they were enthusiastic about using the MI training, and believed that this approach could help children with DCD to be more aware of their motor planning. Therapists reported that children enjoyed this kind of therapy, and that they themselves realized that it is beneficial to teach children new movements from a 1st person perspective. In addition, one therapist reported that the MI training also helped to improve the concentration and focus of the child. When performing this pilot, it appeared that it was difficult and time-consuming to collect all of the measurements (questionnaires, additional video recordings) that were stated in protocol.

## Discussion

The aim of this multiple case study was to explore the feasibility of a recently developed MI-training protocol and to examine individual changes in motor skill in a small-n pilot trial (Adams et al., 2016b). To that end, we used a multi-method evaluation in which we assessed (1) individual changes of motor skills after the training (2) experiences of the children and their parents, and (3) experiences of the therapists. Results showed clinically significant levels of improvement among children in both the MI training and CO-OP training group, but not uniformly. Importantly, results for part C of the MCQ show that both parents and children perceived an improvement in motor ability after training. Indeed, even those children who achieved a minor improvement of 1 standard score on the m-ABC-2 (case 1 and 2 of MI) were perceived by parents (and by the children themselves) as showing improvements in their motor skills (**Figure 2**). This underscores the importance of gathering corroborating data from other sources when assessing change (Hillier, 2007). In the CO-OP group discrepancies between the parents’ and children’s score on part C of the MCQ were found (**Figure 2**). Discrepancies between the children’s and parents’ view on motor proficiency and improvement after training are well known. Kennedy et al. (2013) showed that parents’ views supported the results of standardized assessment and reinforces the value of eliciting parents’ perspectives during the assessment process. It was also found that children’s perspectives did not predict the results of standardized assessments. However, children’s views of their abilities are important because they may have an impact on their motivation to engage in therapy activities. To capture the most comprehensive picture of a child’s motor skills, it is important to combine both standardized assessments (such as the m-ABC-2), parents’ and child’s perspective of motor proficiency (Kennedy et al., 2013). In the current study, children enjoyed both types of training, as indicated by the Enjoyment Scale, which is vital for compliance and general motivation (Barlett and Palisano, 2002). Therapists that provided the MI training were enthusiastic about the therapy, but found it difficult to treat in line with a strict protocol. These results add to the growing body of evidence suggesting that MI training could serve as an adjunct for treatment of motor problems in DCD. The multi-method assessment of the feasibility of MI training in children with DCD in the current study is a necessary and important step before enlisting a full-scale (randomized) clinical trial. The theoretical and applied issues for MI training resulting from this pilot study are discussed below, along with the limitations of this study.

The application of MI training in children raises an important developmental issue that is not apparent in adults, namely the age at which children are first able to enlist MI reliably in the context of treatment (Crajé et al., 2010). Recent developmental data sheds some light on this issue. A review by Spruijt et al. (2015) concluded that MI ability improves steadily over childhood but approaches adult levels by mid-to-late adolescence. These developmental trends supported the view that MI training is feasible for pediatric rehabilitation in typically developing children as young as 5 years of age. However, they suggest that younger children are likely to benefit from MI training when it is presented in an implicit way, and that AO training might be a useful adjunct for this. In line with this, the present results indicate that an MI training, which involved AO can be used as a feasible adjunct in pediatric rehabilitation in children with DCD from 7 years of age.

In the current study, we used a combination of MI, AO and overt practice. AO helped to build a representation of the skill, while overt practice enhanced the development of an internal model of the motor skill. It was hypothesized that this combined approach would promote acquisition of new skills because it trains internal modeling processes by both mental and physical practice (Vogt et al., 2013; Ridderinkhof and Brass, 2015; Di Rienzo et al., 2016). Evidence of improved performance on the m-ABC-2 and perceived benefits of training for new skill learning (reported by parents and children) in the present study lend support for this hypothesis.

Therapists that provided the MI training were enthusiastic about the training, and noted that it helped children with DCD to be more aware of their motor planning. One child with a ADHD diagnosis was included in the MI training group, and the involved therapist reported afterward that the MI training improved the focus and attentional span of this child. Therefore, it is suggested that also children with ADHD may benefit from MI training, and a ADHD diagnosis should not be used *a priori* as an exclusion criteria for these kind of trainings. Negative comments of the therapists were that it was hard to stick to the guidelines of the protocol and that filling in the required forms was time-consuming. This latter finding was not only true for the MI training, but also for the CO-OP training. The research protocol yielded extra (administrative) work for the therapists although we tried to limit this burden. Future work should try to decrease the extra effort of therapists even further, for example by introducing online surveys that could replace the paper forms. Furthermore, our feasibility study shows that is important to closely monitor adherence to protocol’s guidelines during such a study.

A limitation of evaluating training effects with standardized tests is that many of the tasks trained are not measured with the m-ABC-2 (Henderson et al., 2007). Therefore, far transfer, and not improvement of the trained motor skill is measured with this test. However, most of the trained motor skills were present in the MCQ. Ideally, the improvement of specifically the trained motor skill should also be evaluated. In future studies video-recordings of the performance of the two trained motor skills during the first and last treatment could therefore be systematically evaluated. An important next step is to extend the promising findings with a larger clinical trial, incorporating a systematic evaluation of near and far transfer effects.

In sum, this study demonstrated the feasibility of our treatment protocol (Adams et al., 2016b) for use with a clinical group of school-age children with DCD and results extend earlier efficacy studies (Wilson et al., 2002, 2016a). Importantly, this was demonstrated across different levels of evaluation: behavioral measures of movement skill, clinical implementation and questionnaires addressing usability and skill acquisition. In line with earlier research (e.g., Miller et al., 2001; Polatajko et al., 2001; Corcoran et al., 2005) the CO-OP approach also resulted in improved motor skills in children with DCD (perceived improvement and improvement on standardized test). A critical question to be addressed in future work is whether MI protocols can be integrated within traditional physical or occupational therapy as a cost effective adjunct to therapy; this might take the form of home-based training using tablet PCs and other technologies; e.g., a suite of training videos accessed online.

## Author Contributions

IA, BS-E, JL, PW, and BS contributed to the conception and design of the trial. BS-E and IA developed the material for the MI training, and provided training for participating pediatric physical and occupational therapists. IA, BS-E, JL, and BS helped in the acquisition of participants and analyses of results. IA helped in data collection and drafted the manuscript. BS-E, JL, PW, and BS critically reviewed several versions of the manuscript. All authors agree with publication of the final version of the manuscript.

## Conflict of Interest Statement

The authors declare that the research was conducted in the absence of any commercial or financial relationships that could be construed as a potential conflict of interest.
